# Cytotoxic Activity of Fatty Acids From Antarctic Macroalgae on the Growth of Human Breast Cancer Cells

**DOI:** 10.3389/fbioe.2018.00185

**Published:** 2018-12-03

**Authors:** Bruna Silveira Pacheco, Marco Aurélio Ziemann dos Santos, Eduarda Schultze, Rosiane Mastelari Martins, Rafael Guerra Lund, Fabiana Kömmling Seixas, Pio Colepicolo, Tiago Collares, Favero Reisdorfer Paula, Claudio Martin Pereira De Pereira

**Affiliations:** ^1^Bioforensic Research Group, Lipidomic and Bio-Organic Laboratory, Postgraduate Program in Biochemistry and Bioprospection, Federal University of Pelotas, Pelotas, Brazil; ^2^Research Group on Cellular and Molecular Oncology, Postgraduate Program in Biotechnology, Federal University of Pelotas, Pelotas, Brazil; ^3^Chemistry Institute, University of São Paulo, São Paulo, Brazil; ^4^Laboratory of Research and Drugs Development, Pharmaceutical Sciences Postgraduate Program, Federal University of Pampa, Bagé, Brazil

**Keywords:** South Shetland Islands, Antarctic, macroalgae, fatty acid, steroid, polyunsaturated fatty acids, breast cancer

## Abstract

Macroalgae are a natural source of clinically relevant molecules such as polyunsaturated and monounsaturated fatty acids. The Antarctic environment, due to its cold climate, leads to high production of these bioactive molecules. *Adenocystis utricularis, Curdiea racovitzae*, and *Georgiella confluens* from three distinct islands in the Antarctic Peninsula were collected and analyzed for their fatty acid content by gas chromatography flame ionization detection. Results revealed that the algal extracts consisted of 22 fatty acids, of which 9 were saturated, 4 were monounsaturated, and 9 were polyunsaturated (PUFA). In addition, fucosterol was identified within the lipidic extracts. The cytotoxic activity of these fatty acids was evaluated in human breast cancer cell lines MCF-7 and MDA-MB-231. The most notable result was the effect of PUFA on the growth inhibition of cancer cells ranging from 61.04 to 69.78% in comparison to control cells. Significant cytotoxic activity of fatty acids from *A. utricularis* was observed at 48 h, resulting in an inhibition of growth of more than 50% for breast cancer cells at a concentration of 100 μg/mL. A cell viability assay showed that the fatty acids from *A. utricularis* significantly reduced cell viability (68.7% in MCF-7 and 89% in MDA-MB-231 after 72 h of exposure). At the same time, DAPI staining demonstrated chromatin condensation, and apoptotic bodies formed in cells that were cultured with fatty acids from *A. utricularis*. These data indicate that fatty acids from Antarctic macroalgae have the potential to reduce the proliferation of and induce apoptosis in breast cancer cells.

## Introduction

Marine natural products (MNP) exhibit a wide range of biological activities, including antidiabetic, antimicrobial, anti-inflammatory, antiviral, and anticancer effects (Mayer et al., 2013; Petit and Biard, 2013). Currently, more than 22,000 molecules from the marine environment have been reported (Petit and Biard, 2013). These MNP are derived from organisms such as fungi, bacteria, sponges, coral, as well as micro- and macroalgae. The main classes of bioactive molecules found in marine organisms are terpenes, alkaloids, peptides, toxins, and lipids. Moreover, new compounds isolated from marine sources often lead to the discovery of molecules with a variety of clinical and industrial applications (Blunt et al., 2015).

Algae comprise a polyphyletic group of photosynthetic organisms, separated into the phyla Ocrophyta, Chlorophyta, and Rhodophyta, and possess excellent capacity to adapt to extreme environmental conditions. For this reason, and in contrast to terrestrial plants, algae are able to produce a wide variety of unusual compounds such as phlorotannins (oligomers of phloroglucinol through the acetate–malonate pathway) and specific polysaccharides (e.g., sulfated) (Li et al., 2011; Brown et al., 2014). Among the marine compounds with notable activity, fatty acids (FA), especially omega-3 polyunsaturated fatty acids (*n*-3 PUFA), provide several health benefits for chronic diseases such as cancer (Berquin et al., 2008), hyperlipidemia and coronary heart disease (Jump et al., 2012). Furthermore, our previous studies on algae and plants have shown that natural products from Brazilian red propolis and from Antarctic macroalgae have antitumoral activity (Frozza et al., 2013; Gambato et al., 2014; Martins et al., 2018).

Breast cancer is the most prevalent cancer in women worldwide, for which treatment includes chemotherapy, radiation therapy, and/or surgical intervention (Miller et al., 2016). Most anticancer drugs are relatively ineffective against some phases of tumorigenesis, encouraging the search for new alternatives in breast cancer treatment (Zanoaga et al., 2018). Epidemiological studies have shown that PUFA may reduce the incidence of several diseases such as cancer (Gerber, 2012). Additionally, recent studies have revealed that *n*-3 PUFA have an inhibitory role in the development and progression of breast cancer (D'Archivio et al., 2018; Zanoaga et al., 2018). Though the mechanism of how *n*-3 PUFA affects cancer cell growth is not completely understood, it is thought that these acids may interfere with the cell cycle or increase cell death by necrosis or apoptosis (Corsetto et al., 2011). Moreover, recent studies have shown that *n*-3 PUFA can be incorporated into membrane phospholipids and lipid raft on breast cancer tumor cells, which would presumably lead to changes in the fluidity and structure of the cell membrane (Corsetto et al., 2012). Perturbations in the membrane physiology may alter protein composition or the activities of proteins that serve as ion channels, transporters, receptors, signal transducers, or enzymes (Fabian et al., 2015; VanderSluis et al., 2017).

Although several algal species have been studied in regard to their fatty acid composition, a large number of species remains unexplored (El Gamal, 2010; Garcia-Vaquero and Hayes, 2016). Due to its geographical isolation and climatic conditions, Antarctica is a relatively unexplored source of biodiversity. Approximately 130 species have been identified in Antarctica (Wulff et al., 2009); the major fraction of this diversity is distributed throughout the Antarctic Peninsula and, to a lesser extent, in the region comprising the Ross Sea, at longitude 77°S (Wiencke and Amsler, 2011). In the present study, we determined the FA composition of three Antarctic macroalgal species: *Adenocystis utricularis, Curdiea racovitzae*, and *Georgiella confluens* and evaluated their cytotoxic effects on the growth of human breast cancer cells.

## Materials and methods

### Macroalgae samples

Specimens of macroalgae were collected during low summer tides in December 2013, in the Antarctic Peninsula, South Shetland Islands. The investigated macroalgae and the collected information are listed in Table 1. Samples were washed and manually cleaned with local seawater to remove all extraneous matter, then dried at ambient temperature (~0°C) up to 24 h and placed into plastic bags to protect them from light. Macroalgae were collected from their naturally occurring region: eulittoral for *A. utricularis* (up to 2 meters) and sublittoral for *C. racovitzae* and *G. confluens* (2–10 meters). Around 10 g of wet algal material was harvested to obtain ~1 g of dry biomass.

**Table 1:** Species and data collection of macroalgae used in the study.

**Species**	**Data collection**
*Adenocystis utricularis* (Bory de Saint-Vincent) Skottsberg	Robert (62°22'S, 059°41'W) December 6 2013
*Curdiea racovitzae* Hariot	Livingston, Punta Hannah (62°39'S, 60°36'W) December 5 2013
*Georgiella confluens* (Reinsch) Kylin	King George, Vaureal (62°11′S, 58°18′W) December 10 2013

### Chemicals

FAME 37-Mix (Supelco, Bellefonte, Pennsylvania, USA) was used as the analytical standard, and nonadecanoic acid (C19:0; Sigma-Aldrich, St. Louis, Missouri, USA) was used as an internal standard in FA extraction and identification. The reagents used were analytical grade and HPLC-grade solvents.

### Lipid extraction and fatty acid methyl esters preparation

The dried biomass was ground in its entirety (stipe and blade) in a Wiley Knife Mill (Biotech, model B-602), and 1 g from each milled sample was stirred in 30 mL chloroform/methanol (1:2, by vol) and 10 mL sodium sulfate (1.5 g/L) for 30 min at 20°C with a reflux condenser. After 30 min, 10 mL chloroform and 10 mL sodium sulfate (1.5 g/L) were added. The extracts were centrifuged at 2,500 rpm, and the organic phase (~19.8 mL) was separated and evaporated under vacuum and dried using nitrogen (purity >99.998%) until free of organic phase (~15 min), according to Bligh and Dyer's method (Bligh and Dyer, 1959). Yields of the lipidic extracts ranged from 1 to 2% (w/v). The fatty acids were converted to their methyl esters using the boron trifluoride-methanol (BF_3_) method as previously described in the literature (Moss et al., 1974). The resultant mixture of fatty acid methyl esters (FAME) in hexane/chloroform (4:1, by vol) was subjected to gas chromatography-flame ionization detection (GC-FID).

### Gas-chromatography analysis

The quantitative GC analyses were performed according to the following conditions using a gas chromatograph GC/FID-2010 with an AOC-20i autosampler (Shimadzu Corporation, Kyoto, Japan) equipped with a fused-silica capillary column (Rtx-WAX, 30 m × 0.25 mm I.D. × 0.25 μm film thickness). Injections were performed with a 1:25 split ratio and hydrogen was used as the carrier gas under constant flow mode at 1.2 mL/min. The injector was heated to 250°C, and the flame-ionization detector operated at 250°C. The initial programmed oven temperature was 100°C, which was increased by 7°C/min up to 200°C, increased by 5°C/min to 202.6°C and held isothermal for 2 min at this temperature. It was then increased by 5°C/min to 222.9°C and held isothermal for 2 min, and then increased by 5°C/min to 230°C and held isothermal for 10 min at 230°C (Martins et al., 2016). The internal standard solution, containing nonadecanoate methyl ester (19:0 ≥ 99.0%; Sigma-Aldrich, St. Louis, Missouri, USA), was prepared at a concentration of 2 mg/mL by dissolving 20 mg methyl nonadecanoate in 10 mL of n-hexane in a volumetric flask. Results obtained in similar conditions were also confirmed by gas chromatography-mass spectrometry (GC-MS) (Shimadzu QP-2010). The analyses were performed at the Federal University of Pelotas (Laboratory of Lipidomics and Biorganic).

### Determination of cytotoxicity

#### Cell culture

Human breast cancer adenocarcinoma MCF-7 and MDA-MB-231 and a non-tumoral cell line (CHO) were obtained from the Rio de Janeiro Cell Bank (PABCAM, Federal University of Rio de Janeiro, Rio de Janeiro, Brazil) and cultured routinely in our laboratory. The MCF-7 and CHO cells were cultured in Dulbecco's modified Eagle's medium (DMEM), supplemented with 10% (v/v) fetal bovine serum (FBS) purchased from Vitrocell Embriolife (Campinas, Brazil) and Gibco (Grand Island, NY, USA), respectively. The MDA-MB-231 cells were cultured in a LEIBOVITZ L-15 medium (purchased from Cultilab, Campinas, São Paulo, Brazil) supplemented with 10% (v/v) FBS, 1% (v/v) *L*-glutamine, 1% (v/v) penicillin, and 0.2 mg/mL sodium bicarbonate. The cells were seeded at a density of 2 × 10^4^ cells per well in a volume of 100 μL in 96-well plates, and grown for 24 h at 37°C, in an atmosphere of 95% humidified air and 5% CO_2_ (with the exception of MDA-MB-231, which was grown without any CO_2_). The experiments were performed with cells in the logarithmic phase of growth. Cells without any treatment were used as controls in all the experiments. To evaluate the most promising macroalgae, we compared the *A. utricularis, C. racovitzae*, and *G. confluens* macroalgae antiproliferative effect against the breast cancer cell lines (MDA-MB-231 and MCF-7) and the normal cell line (CHO) using their IC_50_ value. The IC_50_ represents the lowest concentration tested capable of inhibiting 50% of the cellular growth.

#### Cell viability assay

The viability of the MCF-7, MDA-MB-231 and CHO cell lines was assessed by MTT (3-(4,5-dimethylthiazol-2-yl)-2,5 diphenyltetrazolium bromide). The cells were treated with different concentrations of FA (1–200 μg/mL) from the macroalgae *A. utricularis, C. racovitzae*, and *G. confluens* for 24, 48, and 72 h. These components were dissolved in dimethyl sulfoxide (DMSO) and added to the medium supplemented with 10% FBS (v/v) at the desired concentrations. The final DMSO concentration in the medium did not exceeded 0.2% (v/v). Control groups that were treated with an equivalent volume of the solvent or containing only the medium were also included.

Thereafter, the incubation medium was removed and 180 μL of medium and 20 μL MTT (5 mg MTT/mL solution) were subsequently added to each well. The plates were incubated for an additional 3 h, and the medium was discarded. DMSO was added to each well, and the formazan was solubilized on a shaker for 5 min at 100 rpm. The absorbance was read on a microplate reader (Victor X5, PerkinElmer, USA) at a test wavelength of 492 nm. Cell inhibitory growth was determined as follows: cell growth inhibition ratio = (1 – Abs_492treated_ _cells_/Abs_492controlcells_) x 100%. For the control group of cells, DMSO and FA were not added. All the observations were validated by at least two independent experiments in triplicate for each experiment.

#### LIVE/DEAD assay

The LIVE/DEAD Cell Viability Assay® (Invitrogen™, Carlsbad, CA, USA) was performed according to the manufacturer's instructions for the most significant result observed from the MTT assay. Briefly, the cells were treated with 100 μg/mL of FA extracted from macroalgae *A. utricularis* for 48 h. The live cells were able to take up calcein and could be analyzed by green fluorescent light emission (488 nm). Ethidium bromide homodimer diffuses through the permeable membranes of dead cells and binds to DNA. Dead cells could be detected using a red fluorescent signal (546 nm). The LIVE/DEAD assay was analyzed using an Olympus IX71 fluorescence microscope (Olympus Optical Co.) with multicolor imaging. The number of red (dead) and green cells (live) in a total of 300 cells was counted in triplicate for each sample. Viability was expressed as the average percentage of viable cells. This experiment was repeated two times.

#### DAPI assay

Apoptosis was detected using DAPI (4′,6-diamidino-2-phenylindole) staining, which forms a fluorescent complex with double-stranded DNA. Cells seeded in a 96-well plate were treated with 100 μg/mL of FA extracted from *A. utricularis* macroalgae. After incubation, the cells were washed three times in phosphate-buffered saline (PBS) and fixed with 1 mL methanol solution at room temperature for 10 min. The fixed cells were then washed with PBS and stained with a DAPI solution at room temperature in the dark. The nuclear morphology of the cells was examined by fluorescence microscopy with an Olympus IX71 (Olympus Optical Co.).

### Statistical analysis

The data in the histogram were previously analyzed to evaluate the distribution, and with the data in a normal distribution, the following tests were performed: MTT assay datasets were analyzed using a factorial ANOVA followed by a Tukey test for multiple comparisons, while the LIVE/DEAD and DAPI assays were analyzed using two-way ANOVA followed by Bonferroni's *post hoc* test. The independent variables were “concentration” and “time.” The dependent variable was the cell growth inhibition ratio (%). IC_50_ values were obtained using GaphPad Prism 5 software. All the data were expressed as mean ± SEM. Significance was considered at *p* value < 0.05 in all the analyses.

## Results and discussion

### Fatty acid composition of macroalgae *A. utricularis, C. racovitzae*, and *G. confluens*

FAs from macroalgae samples were identified and quantified using GC-FID analyses. The total FA content in the analyzed algae is shown in Table 2. A total of 20 different FAs were identified from the Ochrophyta algae *A. utricularis*, whereas 15 and 13 different FAs were identified from the Rhodophyta macroalgae *C. racovitzae* and *G. confluens*, respectively. The results presented throughout this work are expressed as mole percent (number of molecules of one component divided by the total number of molecules in the mixture x 100).

**Table 2:** Fatty acid composition of Antarctic macroalgae.

**Fatty acid**	**Concentration (%)**		
	***Adenocystis utricularis***	***Curdiea racovitzae***	***Georgiella confluens***
Lauric acid (12:0)	–	1.33 ± 0.00	–
Myristic acid (14:0)	6.46 ± 0.01	1.85 ± 0.01	2.24 ± 0.02
Pentadecanoic acid (C15:0)	0.23 ± 0.00	0.24 ± 0.00	0.34 ± 0.00
Palmitic acid (C16:0)	19.15 ± 0.04	20.62 ± 0.11	21.77 ± 0.20
Palmitoleic acid (C16:1)	0.39 ± 0.00	1.39 ± 0.00	5.28 ± 0.03
Heptadecanoic acid (C17:0)	0.51 ± 0.00	0.49 ± 0.00	–
Heptadecenoic acid (17:1)	0.71 ± 0.00	–	–
Stearic acid (C18:0)	2.19 ± 0.01	2.92 ± 0.02	1.41 ± 0.00
Oleic acid (C18:1*n*9c)	8.25 ± 0.01	1.26 ± 0.00	1.29 ± 0.01
Linoleic acid (C18:2*n*6c)	9.36 ± 0.01	1.56 ± 0.02	1.30 ± 0.00
γ-Linolenic acid (C18:3*n*6)	0.55 ± 0.00	0.86 ± 0.00	0.78 ± 0.00
α-Linolenic acid (C18:3*n*3)	9.99 ± 0.02	–	3.05 ± 0.01
Arachidic acid (C20:0)	0.52 ± 0.00	–	–
cis-11,14-Eicosadienoic acid (C20:2*n*6)	0.27 ± 0.00	0.10 ± 0.00	–
cis-8,11,14-Eicosatrienoic acid (C20:3*n*6)	0.81 ± 0.00	1.10 ± 0.00	0.53 ± 0.00
Arachidonic acid (C20:4*n*6)	9.05 ± 0.00	15.06 ± 0.08	1.70 ± 0.01
cis-11,14,17-Eicosatrienoic acid (C20:3*n*3)	0.45 ± 0.00	–	–
Eicosapentaenoic acid (C20:5*n*3)	30.56 ± 0.03	51.10 ± 0.26	59.39 ± 0.54
Behenic acid (C22:0)	0.35 ± 0.00	–	–
Docosahexaenoic acid (C22:6*n*3)	–	–	0.93 ± 0.01
Lignoceric acid (C24:0)	0.04 ± 0.00	–	–
Nervonic acid (24:1*n*9)	0.11 ± 0.00	0.08 ± 0.00	–
Σ*n*-3	41.0	51.10	63.37
Σ*n*-6	20.04	18.68	4.31

*Σ: Sum of all n-3 or n-6 founded to each alga. Fatty acid composition was determined by GC-FID analysis. Data, expressed as mole percent, are means of 3 independent experiments ± SD. – Not detected*.

Saturated fatty acids (SFAs) represented 29.45% of the total FA count in the macroalgae *A. utricularis*. In the red algae *C. racovitzae* and *G. confluens*, the total percentages of SFA were 27.45 and 25.76%, respectively. The most abundant SFA in all the macroalgae studied was palmitic acid (16:0). The largest concentrations of the monounsaturated fatty acids (MUFAs), oleic acid (8.25% of total FA), and palmitoleic acid (5.28% of total FA) were found in *A. utricularis* and *G. confluens*, respectively. PUFA constituted more than half of the total FA in the studied macroalgae, ranging from 61.04 to 69.78% of the total FA. The highest concentrations of linoleic acid (9.36% of total FA) and linolenic acid (9.99% of total FA) were found in the red macroalgae *A. utricularis*. Among the unsaturated FAs, eicosapentaenoic acid (EPA, 20:5*n*-3) was the most representative PUFA in all the species (ranging from 30.56 to 59.39% of the total FA).

Some FAs were found only in *A. utricularis*, such as heptadecenoic acid (17:1), *cis*-11,14,17-eicosatrienoic acid (20:3*n*3), behenic acid (22:0), and lignoceric acid (24:0), corresponding to 0.71, 0.45, 0.35, and 0.04% of the total FA, respectively. Moreover, lauric acid (12:0, 1.33% of total FA) and docosahexaenoic acid (22:6*n*3, 0.93% of total FA) were only found in *C. racovitzae* and *G. confluens*, respectively. The proportion of fatty acids can be seen in Table 2.

The biosynthesis of fatty acids in macroalgae is likely to change according to environmental conditions. Kumari and co-authors analyzed the fatty acid composition of 27 tropical macroalgae (Kumari et al., 2010) and found that only 5 species displayed a PUFA concentration higher than 50% of total fatty acids (values ranged from 14.7 to 62.7%). In our study, the high PUFA values found in these Antarctic macroalgae (ranging from 61.04 to 69.78%) can be explained by their necessity for adapting to temperatures lower than −20°C (Thomas and Dieckmann, 2002). To regulate membrane fluidity in response to low temperatures, macroalgae and microalgae from cold regions produce large amounts of FA and increase the degree of unsaturation as a result of adaptation to the environment, producing PUFA in larger quantities, especially *n*-3 (Jiang and Chen, 2000; Graeve et al., 2002; Bhosale et al., 2009). In this way, the physiological role of the lipid membrane is maintained (Los et al., 2013). FAs are an important class of lipids, which act as energy-storage molecules and components of cell membranes, and are commonly present as esters of glycerol in phospholipids, glycolipids and triacylglycerols. Moreover, they act as intra/extracellular messengers and as cofactors in enzymatic electron transfer reactions in mitochondria and chloroplasts. In recent years, our research group has identified FAs from macroalgae (Martins et al., 2016; Pereira et al., 2017; Santos et al., 2017) and synthetic derivatives (Hobuss et al., 2012; Pacheco et al., 2014).

Macroalgae from the Ochrophyta phylum are known to have higher concentrations of C20 PUFA than C18 PUFA, which is in agreement with our results for the brown macroalgae *A. utricularis* (Narayan et al., 2005). In this study, the total content of C20 PUFA in brown algae was 41.14%, while the total C18 PUFA was 19.90%. The main C20 PUFA shown was eicosapentaenoic acid (20:5*n*3), ranging from 30.56 to 59.39% of the total number of FAs. In addition to these FAs, Kumari and co-authors (Kumari et al., 2010) found large quantities of myristic acid (14:0) and palmitic acid (16:0) in brown macroalgae. This is similar to our findings with myristic acid and palmitic acid, comprising 6.46 and 19.15% of the total FA, respectively, in *A. utricularis*.

Among red algae (Rhodophyceae), the literature describes that the most prevalent PUFAs are arachidonic acid (ARA, 20:4*n*6) and EPA (20:5*n*3) (Kumari et al., 2010), which is in agreement with our results, with the exception of *G. confluens*, which presented ARA corresponding to 1.70% of the total FAs. The FA composition of Antarctic *G. confluens* was previously studied by Graeve et al. (2002). In agreement with our study, *G. confluens* was rich in palmitic acid and eicosapentaenoic acid, but in the present study, we found the highest level of total *n*-3 PUFAs in *G. confluens*. The variation in FA content between species of the same phylum can be influenced by season, geographical region, and age (Thomas and Dieckmann, 2002). Palmitic acid (16:0) was the most prevalent SFA in all the red algae studied (20.62 to 21.77% of the total FA). The high values of *n*-3 found in the macroalgae under study are relevant because, unlike mammals, algae are able to produce PUFAs such as n-3 and n-6, which permits survival at low temperatures, and varying levels of solar irradiation (Wiencke et al., 2007).

### Determination of cell proliferation

**Figure 1:**
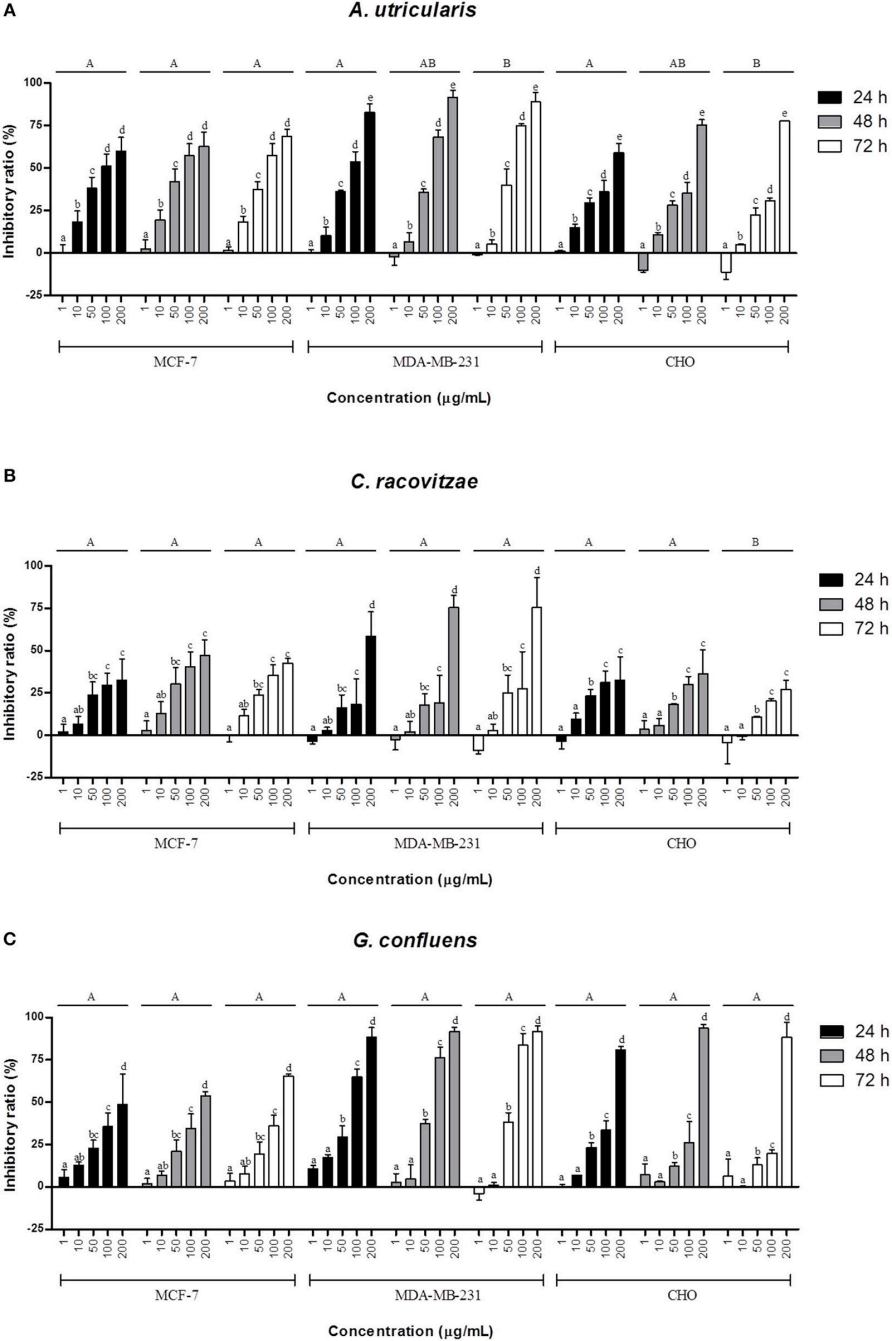
Effects of different concentrations of the combination of FAs extracted from macroalgae. **(A)** *A. utricularis* **(B)** *C. racovitzae* and **(C)** *G. confluens* at 24, 48, and 72 h in inhibiting MCF-7, MDA-MB-231, and CHO cells. Cytotoxicity was assessed by MTT assay. The data are expressed as the mean ± SEM of a representative experiment performed in duplicate (*n* = 2). Significance was considered at *p* < 0.05 (Tukey test). Capital letters indicate differences between the different times. The lowercase letters indicate differences between the different concentrations. Differences between cells were not considered.

The extracted FAs from Antarctic macroalgae were evaluated for cytotoxic activity against MCF-7 and MDA-MB-231cells. The combination of FA extracted from each alga was diluted to the following tested concentrations: 1, 10, 50, 100, and 200 μg/mL. Fatty acid extract from macroalgae *A. utricularis* demonstrated significant activity at 48 h, inhibiting the growth of more than 50% of tumor cells at a concentration of 100 μg/mL. Non-tumoral cells did not reach 50% growth inhibition, with the exception of macroalgae *A. utricularis* and *G. confluens*, which achieved 50% inhibition at the highest concentration (200 μg/mL). For all the tumor and non-tumor cells analyzed, cell viability decreased in a concentration- and time-dependent manner (Figures 1A–C). The *A. utricularis* macroalgae displayed the lowest IC_50_ value in MCF-7 when compared to the other magroalgae at all times tested (Table 3). In addition, it also demonstrated a satisfactory antiproliferative effect in MDA-MB-231 and selectivity in its cytotoxicity, since the *A. utricularis* macroalgae demonstrated a higher IC_50_ value in the normal cell line than the tumoral ones.

**Table 3:** IC_50_ ± SEM values of the *A. utricularis, C. racovitzae* and *G. confluens* macroalgae related to time of exposure (24, 48, and 72 h) and the different cell lines (MCF-7, MDA-MB-231, and CHO).

	**24 h**			**48 h**			**72 h**		
	***A. utricularis***	***C. racovitzae***	***G. confluens***	***A. utricularis***	***C. racovitzae***	***G. confluens***	***A. utricularis***	***C. racovitzae***	***G. confluens***
MCF-7	90.42 ± 30.53	N/A	164.4 ± 64	72.92 ± 25.64	145.6 ± 58.09	157.5 ± 30.95	74.99 ± 16.46	N/A	134.1 ± 21.35
MDA	77.32 ± 12.43	175.2 ± 32.9	72.57 ± 18.77	66.7 ± 7.47	144.8 ± 35.9	61.5 ± 7.63	59.75 ± 7.63	128.5 ± 26.28	58.78 ± 5.96
CHO	132 ± 40.07	N/A	116.1 ± 19.91	114.2 ± 30.5	N/A	117.6 ± 13.3	123.3 ± 25.55	N/A	127.1 ± 17.85

*N/A–not applicable (it was not possible to calculate the IC_50_ value because the higher concentration tested did not inhibit 50% of the cellular growth)*.

**Figure 2:**
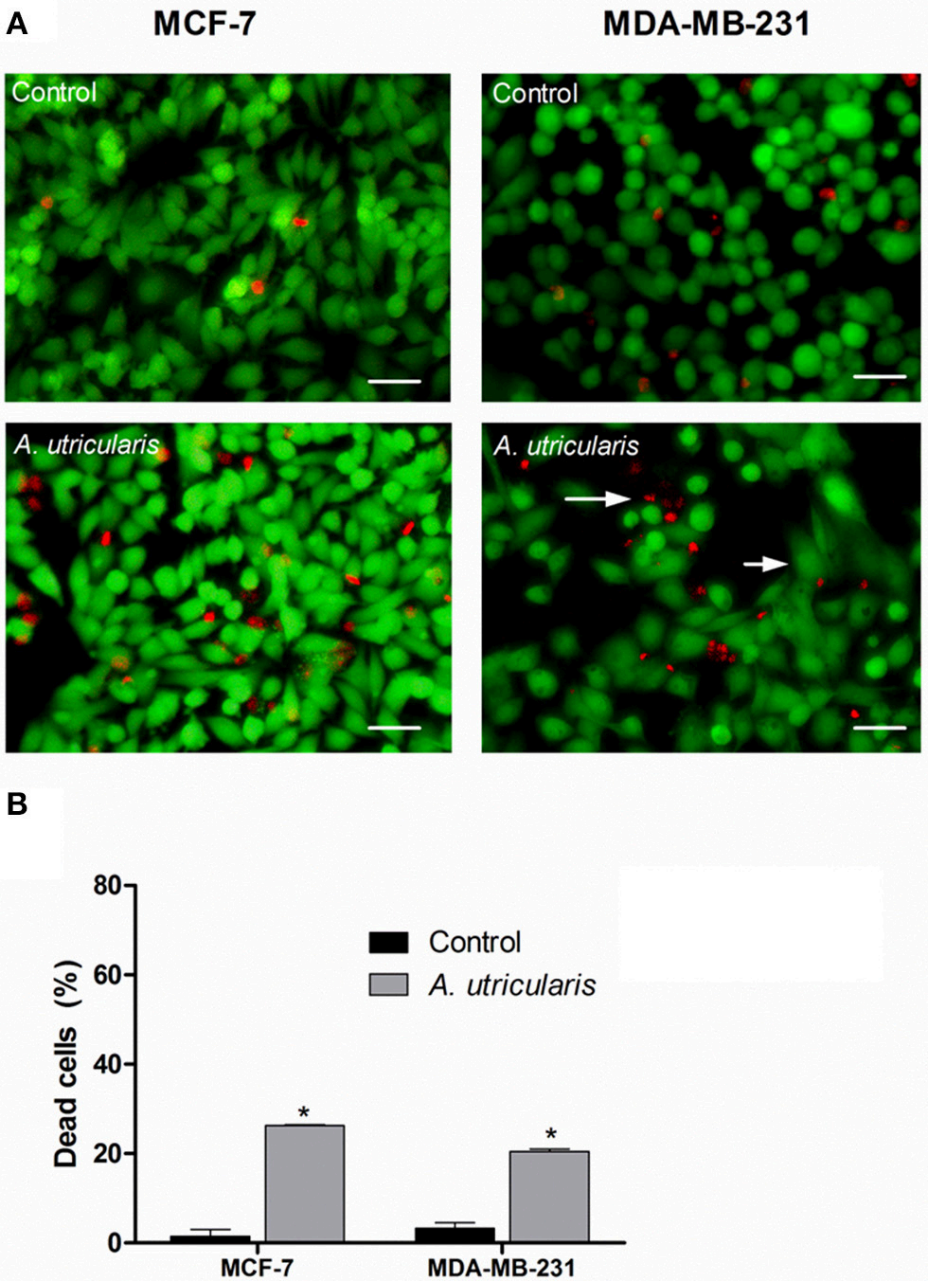
Breast cancer cells were treated with FAs from *A. utricularis* for 48 h. **(A)** The number of cell deaths was estimated by LIVE/DEAD assay, using an Olympus IX71 fluorescence microscope (Olympus Optical Co., Tokyo, Japan). **(B)** The graphic shows the mean ± SEM of three different areas of the plate. The long arrows indicate dead cells (red), and the short arrows indicate live cells (green). The symbols indicate statistical differences. ^*^p < 0.05. The bar indicates 100 μm.

The LIVE/DEAD assay confirmed the cytotoxic effect of FA acids, via fluorometric analysis. The LIVE/DEAD assay showed that the FA from the Antarctic macroalgae *A. utricularis* significantly reduced cell viability in both MCF-7 and MDA-MB-231 cell lines (top panel in Figure 2). The bottom panel in Figure 2 shows the mean number of dead cells calculated from three different areas of the plate. The data demonstrated a significant increase in cell death after treatment with *A. utricularis* compared to the negative control group by the evaluation of live (green fluorescence) and dead cells (red fluorescence).

SFA and MUFA are known to increase cancer risk, while PUFA, specifically *n*-3, is known to possess cytotoxic activity in many cancer types, such as prostate (Gu et al., 2013), colorectal (Song et al., 2014) among others. One of the pathways by which SFA can promote cancer development is through the activation of Toll-like receptors (TLRs), which are important regulators of the innate immune system. SFA can activate TLRs linked to proinflammatory activity, promoting tumor activation, while PUFA can inhibit these TLRs (Gu et al., 2013). Studies suggest that *n*-6 PUFA accelerates tumorigenesis; in contrast, the *n*-3 PUFA may have anticancer effects. While *n*-6 PUFA is found abundantly in meats, vegetable oils, fruits, and a wide variety of foods consumed daily, the longer chain *n*-3 PUFA, such as EPA, can be obtained directly from marine sources.

In addition to fatty acids, sterol composition was also found to vary significantly in different macroalgae. In this study, the algal lipidic extracts were found to be enriched with 4.97% fucosterol and 0.53% avenestrol. Steroids are important structural components of algal cell membranes, due to their amphipathic nature, and exert a substantial physiological function on regulating membrane fluidity and permeability (Kumari et al., 2013). Interestingly, various sterol components are emerging as popular agents for cancer-related therapy. Recently, Kazlowska reported the *in vitro* and *in vivo* anticancer effects of sterol fractions from the red algae *Porphyra dentate*, attributed most likely to the presence of β-sitosterol and campesterol (Kazłowska et al., 2013). In this way, Khanavi et al. reported the cytotoxicity of fucosterol-containing fraction of marine algae against breast and colon carcinoma cell lines. The study indicated that fucosterol, the most abundant phytosterol in brown algae, is responsible for the cytotoxic effect of this extract against breast and colon carcinoma cell lines (Khanavi et al., 2012).

In this study, according to the MTT assay (Figure 1), the extracted FA from all the tested macroalgae showed antitumor activity against both cells, and the inhibition was lower for the non-tumoral CHO cell line than for the breast cancer cell lines MCF-7 and MDA-MB-231. Previous *in viv*o and *in vitro* studies on breast cancer cell lines have shown that *n*-3 PUFA may be effective in reducing cell growth, reducing tumor volume and preventing metastasis, among other benefits, by multiple mechanisms such as influences on transcription factor activity, gene expression, and signal transduction; the alteration of estrogen metabolism; alterations in the production of free radicals and reactive oxygen species; the suppression of neoplasic transformation; the inhibition of cell growth; increased apoptosis; and the inhibition of eicosanoid production from *n*-6 FA precursors (Larsson et al., 2004; Wannous et al., 2013; Liu et al., 2018). The FA tested in this study resulted in little or no cytotoxicity against the CHO cell line at concentrations up to 100 μg/mL. Furthermore, the FAs from the Antarctic macroalgae were generally found to be selective for tumorigenic cells.

Besides reducing tumor growth, FA may also increase the efficacy of radiotherapy and several drugs, such as doxorubicin, epirubicin, 5-fluorouracil, and mitomycin C (Hardman, 2004). FA in combination with Tamoxifen, an anti-estrogen drug, has great clinical potential for reducing breast cancer. An earlier study had shown an improved response with use of this drug and inhibition of the early stages of carcinogenesis (Manni et al., 2014). Though the mechanism has yet to be elucidated, the effects of PUFA may be further complicated by the involvement of genetic factors (Zou et al., 2014).

One of the most notable differences between *A. utricularis* and the other algae studied is the presence of alpha-linolenic acid (ALA) (18:3*n*3), of which 9.99% was found in *A. utricularis*, 3.05% was found in *G. confluens* and none was found in *C. racovitzae*. An earlier study demonstrated that ALA has the capacity to reduce the growth of tumors and induce apoptosis in different breast cancer cell lines (MCF-7, MDA-MB-231, BT-474, and MDA-MB-468) (Wiggins et al., 2015). Linoleic (LA) and oleic acid (OA) also showed higher concentrations in *A. utricularis* (9.36% to LA, 8.25% to OA) than did *C. racovitzae* or *G. confluens*. These FA may inhibit the cancer via multiple mechanisms, including the modulation of peroxisome proliferator-activated receptors (PPAR α, γ, δ), the reduction of *n*-6 fatty acid arachidonic acid release from cell membranes, inhibition of enzymatic activities, and direct competition with arachidonic acid for enzymatic conversions (Wendel and Heller, 2009). Furthermore, ALA, LA, and OA have been cited as modulators of PPARγ receptor and gene activation in MCF7 and MDA-MB-231 cell lines, which attenuate tumor growth and also induce apoptosis (Crowe and Chandraratna, 2004). The higher concentrations of these FAs in *A. utricularis* may explain the higher toxicity observed for breast tumor cell lines in comparison to *C. racovitzae* and *G. confluens*.

Moreover, *A. utricularis* showed the lowest ratio of *n*-6/*n*-3 (2/1) in comparison with *C. racovitzae* (2.7/1) and *G. confluens* (14.7/1). It is known that a low ratio between *n*-6 and *n*-3 is associated with beneficial effects in many diseases (Simopoulos, 2008). However, it is not possible to state that only one fatty acid is responsible for the potential anti-proliferative activity shown by *A. utricularis*, or if a group of FAs plays a synergic effect.

Nevertheless, the percentage of non-viable cells was evaluated by LIVE/DEAD assay (Figure 2). The results showed that treatment with FA from *A. utricularis* at a concentration of 100 μg/mL was effective in inhibiting the growth of tumor cells, resulting in significantly lower numbers of viable cells after 48 h compared to untreated control cells. In MCF-7 and MDA-MB-231, the percentage of non-viable cells due to *A. utricularis* was 26.26 and 21%, respectively, compared to 1.5% of the control cells.

In the literature, several studies have investigated the cytotoxicity induced by FA, mainly *n*-3 PUFA in some types of cancer, such as breast (Kim et al., 2009), colon (Kato et al., 2007), skin (Rhodes et al., 2003), gastrointestinal (Park et al., 2013), prostate (Gu et al., 2013), and pancreatic cancer (Mohammed et al., 2012). While SFA and MUFA have been found to increase the risk of cancer, *n-3* PUFA could be effective for treating the side effects of cancer, such as cachexia, cognitive impairment, distress, pain, and fatigue (Pottel et al., 2014).

Many unexplored resources that have potential for biotechnological applications are present in extremely isolated regions, such as Antarctica. Since climate conditions in Antarctica are variable and algae are exposed to the great seasonal extremes of light and temperature, simulating these growth conditions in the laboratory is not yet a viable alternative. Though past studies on Antarctic macroalgae have mostly been on matters related to physiology, such as photosynthesis and growth (Dhargalkar and Verlecar, 2009), deciphering the metabolite composition of Antarctic macroalgae will be particularly useful for the discovery of new bioactive molecules with clinical applications.

### Determination of apoptosis

**Figure 3:**
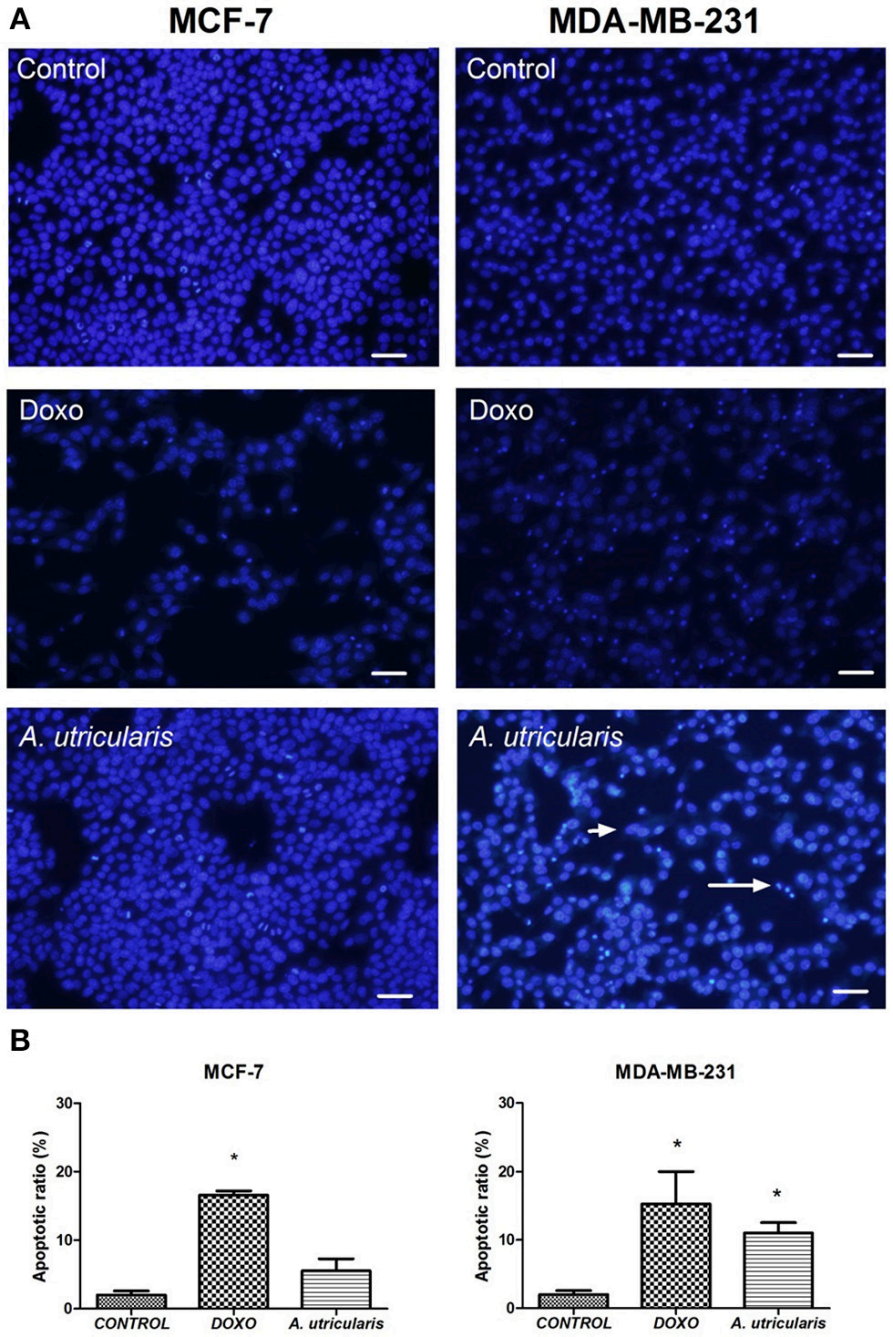
MCF-7 and MDA-MB-231 cell apoptosis (as assessed by DAPI staining). **(A)** The number of apoptotic cells was estimated by DAPI staining, using an Olympus IX71 fluorescence microscope (Olympus Optical Co., Tokyo, Japan). **(B)** The graphic shows the mean ± SEM of three different areas of the plate. The long arrow indicates apoptotic cells (condensed or fragmented nuclei), and the short arrow indicates live cells. The symbols indicate statistical differences. ^*^p < 0.05. The bar indicates 100 μm. DOXO is related to Doxorubicin, the positive control.

The DAPI staining revealed that nuclei with chromatin condensation and apoptotic bodies were formed in cells that were cultured with FA from *A. utricularis* macroalgae, with a significant difference between the sample and control in MDA-MB-231 cells. Figure 3 illustrates the cells stained with DAPI and observed under a fluorescence microscope. Viable cells (control group) with intact DNA and just slightly activated in the fluorescence microscope image were negative for DAPI, and the compound Doxorubicin (0.6 μg/mL) was used as a positive control for DAPI.

Despite the great variability of cancer, evidence shows that resistance to apoptosis is one of the main features of the most malignant tumors (Okada and Mak, 2004). Many studies have demonstrated that PUFA may induce apoptosis in breast cancer cells by the susceptibility of these cells to lipid peroxidation (Schley et al., 2005; Liu et al., 2015); our research group has also been exploring this field (Pereira et al., 2015). The DAPI-staining results suggested that fatty acids from *A. utricularis* induced apoptosis in MDA-MB-231 cells at the tested concentration (Figure 3). Tumor cells have uncontrolled proliferative capacity and an increasing inability to die by apoptosis.

## Conclusions

In summary, the data presented in this work indicated that Antarctic macroalgae produce high amounts of polyunsaturated FA. Moreover, these results demonstrate that FA extracted from this group of macroalgae, particularly *A. utricularis*, were able to inhibit the growth of MCF-7 and MDA-MB-231 human breast cancer cells. Fatty acid extracts from these macroalgae merit further investigation, particularly with regard to their effects on other tumor cell lines as well as in combination with known anti-cancer drugs.

## Author contributions

BP: redactor, lipid extraction and algae collection; MdS: chromatographic analysis; ES: biological tests; RM: lipid extraction; RL: screening with other cell types; FS: biological tests; PC: algae identification; TC: biological assay planning; FP: chemical analysis and medicinal chemistry contributions; CdP: chemical analysis and experiment design.

### Conflict of interest statement

The authors declare that the research was conducted in the absence of any commercial or financial relationships that could be construed as a potential conflict of interest.

## References

[B1] Berquin I. M. Edwards I. J. Chen Y. Q. (2008). Multi-targeted therapy of cancer by omega-3 fatty acids. Cancer Lett. 269, 363–377. 10.1016/j.canlet.2008.03.044 18479809 PMC2572135

[B2] Bhosale R. A. Velankar D. A. Chaugule B. B. (2009). Fatty acid composition of the cold-water-inhabiting freshwater red alga Sirodotia Kylin. J. Appl. Phycol. 21, 99–102. 10.1007/s10811-008-9333-5

[B3] Bligh E. G. Dyer W. J. (1959). A rapid method of total lipid extraction and purification. Can. J. Biochem. Phys. 37, 911–917. 10.1139/y59-099 13671378

[B4] Blunt J. W. Copp B. R. Keyzers R. A. Munro M. H. Prinsep M. R. (2015). Marine natural products. Nat. Prod. Rep. 32, 116–211. 10.1039/c4np00144c 25620233

[B5] Brown E. M. Allsopp P. J. Magee P. J. Gill C. I. Nitecki S. Strain C. R.. (2014). Seaweed and human health. Nutr. Rev. 72, 205–216. 10.1111/nure.12091 24697280

[B6] Corsetto P. A. Cremona A. Montorfano G. Jovenitti I. E. Orsini F. Arosio P.. (2012). Chemical–physical changes in cell membrane microdomains of breast cancer cells after omega-3 PUFA incorporation. Cell Biochem. Biophys. 64, 45–59. 10.1007/s12013-012-9365-y 22622660

[B7] Corsetto P. A. Montorfano G. Zava S. Jovenitti I. E. Cremona A. Berra B.. (2011). Effects of n-3 PUFAs on breast cancer cells through their incorporation in plasma membrane. Lipids Health Dis. 10:73. 10.1186/1476-511X-10-73 21569413 PMC3127786

[B8] Crowe D. L. Chandraratna R. A. (2004). A retinoid X receptor (RXR)-selective retinoid reveals that RXR-alpha is potentially a therapeutic target in breast cancer cell lines, and that it potentiates antiproliferative and apoptotic responses to peroxisome proliferator-activated receptor ligands. Breast Cancer Res. 6, R546–R555. 10.1186/bcr913 15318936 PMC549174

[B9] D'Archivio M. Scazzocchio B. Vari R. Santangelo C. Giovannini C. Masella R. (2018). Recent evidence on the role of dietary PUFAs in cancer development and prevention. Curr. Med. Chem. 25, 1818–1836. 10.2174/0929867325666171204160231 29210633

[B10] Dhargalkar V. K. Verlecar X. N. (2009). Southern Ocean seaweeds: a resource for exploration in food and drugs. Aquaculture 287, 229–242. 10.1016/j.aquaculture.2008.11.013

[B11] El Gamal A. A. (2010). Biological importance of marine algae. Saudi Pharm. J. 18, 1–25. 10.1016/j.jsps.2009.12.001 23960716 PMC3731014

[B12] Fabian C. J. Kimler B. F. Hursting S. D. (2015). Omega-3 fatty acids for breast cancer prevention and survivorship. Breast Cancer Res. 17:62. 10.1186/s13058-015-0571-6 25936773 PMC4418048

[B13] Frozza C. O. Garcia C. S. Gambato G. de Souza M. D. O. Salvador M. Moura S.. (2013). Chemical characterization, antioxidant and cytotoxic activities of Brazilian red propolis. Food Chem. Toxicol. 52, 137–142. 10.1016/j.fct.2012.11.013 23174518

[B14] Gambato G. Baroni É. G. Garcia C. S. Frassini R. Frozza C. O. Moura S. (2014). Brown algae *Himantothallus grandifolius* (Desmarestiales, Phaeophyceae) suppresses proliferation and promotes apoptosis-mediated cell death in tumor cells. Adv. Biol. Chem. 4, 98–108. 10.4236/abc.2014.42014

[B15] Garcia-Vaquero M. Hayes M. (2016). Red and green macroalgae for fish and animal feed and human functional food development. Food Ver. Int. 32, 15–45. 10.1080/87559129.2015.1041184

[B16] Gerber M. (2012). Omega-3 fatty acids and cancers: a systematic update review of epidemiological studies. Brit. J. Nutr. 107, S228–S239. 10.1017/S0007114512001614 22591896

[B17] Graeve M. Kattner G. Wiencke C. Karsten U. (2002). Fatty acid composition of Arctic and Antarctic macroalgae: indicator of phylogenetic and trophic relationships. Mar. Ecol. Prog. Ser. 231, 67–74. 10.3354/meps231067

[B18] Gu Z. Suburu J. Chen H. Chen Y. Q. (2013). Mechanisms of omega-3 polyunsaturated fatty acids in prostate cancer prevention. BioMed Res. Int. 2013:824563. 10.1155/2013/824563 23762859 PMC3676993

[B19] Hardman W. E. (2004). (n-3) fatty acids and cancer therapy. J. Nutr. 134, 3427S−3430S. 10.1093/jn/134.12.3427S 15570049

[B20] Hobuss C. B. Venzke D. Pacheco B. S. Souza A. O. Santos M. A. Moura S.. (2012). Ultrasound-assisted synthesis of aliphatic acid esters at room temperature. Ultrason. Sonochem. 19, 387–389. 10.1016/j.ultsonch.2011.06.020 21940192

[B21] Jiang Y. Chen F. (2000). Effects of temperature and temperature shift on docosahexaenoic acid production by the marine microalge Crypthecodinium cohnii. J. Am. Oil Chem. Soc. 77, 613–617. 10.1007/s11746-000-0099-0

[B22] Jump D. B. Depner C. M. Tripathy S. (2012). Omega-3 fatty acid supplementation and cardiovascular disease Thematic Review Series: new lipid and lipoprotein targets for the treatment of cardiometabolic diseases. J. Lipid Res. 53, 2525–2545. 10.1194/jlr.R027904 22904344 PMC3494243

[B23] Kato T. Kolenic N. Pardini R. S. (2007). Docosahexaenoic acid (DHA), a primary tumor suppressive omega-3 fatty acid, inhibits growth of colorectal cancer independent of p53 mutational status. Nutr. Cancer 58, 178–187. 10.1080/01635580701328362 17640164

[B24] Kazłowska K. Lin H. T. V. Chang S. H. Tsai G. J. (2013). *In vitro* and *in vivo* anticancer effects of sterol fraction from red algae *Porphyra dentata*. Evid. Based Complement. Alternat. Med. 2013:493869. 10.1155/2013/493869 24062783 PMC3770035

[B25] Khanavi M. Gheidarloo R. Sadati N. Ardekani M. R. S. Nabavi S. M. B. Tavajohi S.. (2012). Cytotoxicity of fucosterol containing fraction of marine algae against breast and colon carcinoma cell line. Pharmacogn. Mag. 8, 60–64. 10.4103/0973-1296.93327 22438665 PMC3307205

[B26] Kim J. Lim S. Y. Shin A. Sung M. K. Ro J. Kang H. S.. (2009). Fatty fish and fish omega-3 fatty acid intakes decrease the breast cancer risk: a case-control study. BMC Cancer 9:216. 10.1186/1471-2407-9-216 19566923 PMC2711973

[B27] Kumari P. Kumar M. Gupta V. Reddy C. R. K. Jha B. (2010). Tropical marine macroalgae as potential sources of nutritionally important PUFAs. Food Chem. 120, 749–757. 10.1016/j.foodchem.2009.11.006

[B28] Kumari P. Kumar M. Reddy C. R. K. Jha B. (2013). Algal lipids, fatty acids and sterols in Functional Ingredients From Algae for Foods and Nutraceuticals, ed Dominguez H. (Cambridge: Woodhead Publishing), 87–134.

[B29] Larsson S. C. Kumlin M. Ingelman-Sundberg M. Wolk A. (2004). Dietary long-chain n– 3 fatty acids for the prevention of cancer: a review of potential mechanisms. Am. Journal Clin. Nutr. 79, 935–945. 10.1093/ajcn/79.6.935 15159222

[B30] Li Y. X. Wijesekara I. Li Y. Kim S. K. (2011). Phlorotannins as bioactive agents from brown algae. Process Biochem. 46, 2219–2224. 10.1016/j.procbio.2011.09.015

[B31] Liu J. Abdelmagid S. A. Pinelli C. J. Monk J. M. Liddle D. M Hillyer L. M.. (2018). Marine fish oil is more potent than plant-based n-3 polyunsaturated fatty acids in the prevention of mammary tumors. J. Nutr. Biochem. 55, 41–52. 10.1016/j.jnutbio.2017.12.011 29413488

[B32] Liu Z. Hopkins M. M. Zhang Z. Quisenberry C. B. Fix L. C. Galvan B. M.. (2015). Omega-3 fatty acids and other FFA4 agonists inhibit growth factor signaling in human prostate cancer cells. J. Pharmacol. Exp. Ther. 352, 380–394. 10.1124/jpet.114.218974 25491146 PMC4293432

[B33] Los D. A. Mironov K. S. Allakhverdiev S. I. (2013). Regulatory role of membrane fluidity in gene expression and physiological functions. Photosynth. Res. 116, 489–509. 10.1007/s11120-013-9823-4 23605242

[B34] Manni A. Richie J. P. Xu H. Washington S. Aliaga C. Bruggeman R.. (2014). Influence of omega-3 fatty acids on Tamoxifen-induced suppression of rat mammary carcinogenesis. Int. J. Cancer 134, 1549–1557. 10.1002/ijc.28490 24122252

[B35] Martins R. M. dos Santos M. A. Z. Pacheco B. S. Mansilla A. Astorga-España M. S. Seixas F. (2016). Fatty acid profile of the chlorophyta species from Chile's sub-Antarctic region. Acad. J. Sci. Res. 4, 93–98. 10.15413/ajsr.2015.0154

[B36] Martins R. M. Nedel F. Guimarães V. B. S. da Silva A. F. Colepicolo P. Pereira C. M. P.. (2018). Macroalgae extracts from antarctica have antimicrobial and anticancer potential. Front. Microbiol. 9:412. 10.3389/fmicb.2018.00412 29568291 PMC5852318

[B37] Mayer A. Rodríguez A. D. Taglialatela-Scafati O. Fusetani N. (2013). Marine pharmacology in 2009–2011: Marine compounds with antibacterial, antidiabetic, antifungal, anti-inflammatory, antiprotozoal, antituberculosis, and antiviral activities; affecting the immune and nervous systems, and other miscellaneous mechanisms of action. Mar. Drugs 11, 2510–2573. 10.3390/md11072510 23880931 PMC3736438

[B38] Miller K. D. Siegel R. L. Lin C. C. Mariotto A. B. Kramer J. L. Rowland J. H.. (2016). Cancer treatment and survivorship statistics, 2016. CA Cancer J. Clin. 66, 271–289. 10.3322/caac.21349 27253694

[B39] Mohammed A. Janakiram N. B. Brewer M. Duff A. Lightfoot S. Brush R. S. (2012). Endogenous n-3 polyunsaturated fatty acids delay progression of pancreatic ductal adenocarcinoma in Fat-1-p48Cre/+-LSL-KrasG12D/+ mice. Neoplasia 14, 1249–1246. 10.1593/neo.121508 23308056 PMC3540949

[B40] Moss C. W. Lambert M. A. Merwin W. H. (1974). Comparison of rapid methods for analysis of bacterial fatty acids. Appl. Microbiol. 28, 80–85. 4844271 10.1128/am.28.1.80-85.1974 PMC186596

[B41] Narayan B. Miyashita K. Hosakawa M. (2005). Comparative evaluation of fatty acid composition of different Sargassum (Fucales, Phaeophyta) species harvested from temperate and tropical waters. J. Aquat. Food Prod. Technol. 13, 53–70. 10.1300/J030v13n04_05

[B42] Okada H. Mak T. W. (2004). Pathways of apoptotic and non-apoptotic death in tumour cells. Nat. Rev. Cancer 4:592. 10.1038/nrc1412 15286739

[B43] Pacheco B. S. Nunes C. F. Rockembach C. T. Bertelli P. Mesko M. F. Roesch-Ely M. (2014). Eco-friendly synthesis of esters under ultrasound with p-toluenesulfonic acid as catalyst. Green Chem. Lett. Rev. 7, 265–270. 10.1080/17518253.2014.941950

[B44] Park J. M. Kwon S. H. Han Y. M. Hahm K. B. Kim E. H. (2013). Omega-3 polyunsaturated Fatty acids as potential chemopreventive agent for gastrointestinal cancer. J. Cancer Prev. 18, 201–208. 10.15430/JCP.2013.18.3.201 25337547 PMC4189468

[B45] Pereira C. M. Nunes C. F. Zambotti-Villela L. Streit N. M. Dias D. Pinto E. (2017). Extraction of sterols in brown macroalgae from Antarctica and their identification by liquid chromatography coupled with tandem mass spectrometry. J. Appl. Phycol. 29, 751–757. 10.1007/s10811-016-0905-5

[B46] Pereira C. M. P. Colepicolo P. Souza P. O. (2015). Antitumor Activity of Extracts From Antartic Macroalgae. Saarbrücken: Lambert Academic Publishing.

[B47] Petit K. Biard J. F. (2013). Marine natural products and related compounds as anticancer agents: an overview of their clinical status. Anticancer Agents Med. Chem. 13, 603–631. 10.2174/1871520611313040010 23140351

[B48] Pottel L. Lycke M. Boterberg T. Foubert I. Pottel H. Duprez F. (2014). Omega-3 fatty acids: physiology, biological sources and potential applications in supportive cancer care. Phytochem. Rev. 13, 223–244. 10.1007/s11101-013-9309-1

[B49] Rhodes L. E. Shahbakhti H. Azurdia R. M. Moison R. M. Steenwinkel M. J. Homburg M. I.. (2003). Effect of eicosapentaenoic acid, an omega-3 polyunsaturated fatty acid, on UVR-related cancer risk in humans. An assessment of early genotoxic markers. Carcinogenesis 24, 919–925. 10.1093/carcin/bgg038 12771037

[B50] Santos M. A. Colepicolo P. Pupo D. Fujii M. T. Pereira C. M. Mesko M. F. (2017). Antarctic red macroalgae: a source of polyunsaturated fatty acids. J. Appl. Phycol. 29, 759–767. 10.1007/s10811-016-1034-x

[B51] Schley P. D. Jijon H. B. Robinson L. E. Field C. J. (2005). Mechanisms of omega-3 fatty acid-induced growth inhibition in MDA-MB-231 human breast cancer cells. Breast Cancer Res. Treat. 92, 187–195. 10.1007/s10549-005-2415-z 15986129

[B52] Simopoulos A. P. (2008). The importance of the omega-6/omega-3 fatty acid ratio in cardiovascular disease and other chronic diseases. Exp. Biol. Med. 233, 674–688. 10.3181/0711-MR-311 18408140

[B53] Song M. Chan A. T. Fuchs C. S. Ogino S. Hu F. B. Mozaffarian D. (2014). Dietary intake of fish, ω-3 and ω-6 fatty acids and risk of colorectal cancer: a prospective study in US men and women. Int. J. Cancer 135, 2413–2423. 10.1002/ijc.28878 24706410 PMC4159425

[B54] Thomas D. N. Dieckmann G. S. (2002). Antarctic sea ice–a habitat for extremophiles. Science 295, 641–644. 10.1126/science.1063391 11809961

[B55] VanderSluis L. Mazurak V. C. Damaraju S. Field C. J. (2017). Determination of the relative efficacy of eicosapentaenoic acid and docosahexaenoic acid for anti-cancer effects in human breast cancer models. Int. J. Mol. Sci. 18:2607. 10.3390/ijms18122607 29207553 PMC5751210

[B56] Wannous R. Bon E. Mahéo K. Goupille C. Chamouton J. Bougnoux P.. (2013). PPARβ mRNA expression, reduced by n– 3 PUFA diet in mammary tumor, controls breast cancer cell growth. Biochim. Biophys. Acta Mol. Cell Biol. Lipids 1831, 1618–1625. 10.1016/j.bbalip.2013.07.010 23906790

[B57] Wendel M. Heller A. R. (2009). Anticancer actions of omega-3 fatty acids – current state and future perspectives. Anticancer Agents Med. Chem. 9, 457–470. 10.2174/1871520610909040457 19442044

[B58] Wiencke C. Amsler C. D. (2011). Seaweeds and their communities in polar regions, in Seaweed Biology: Novel Insights Into Ecophysiology, Ecology and Utilization, eds Wiencke C. Bischof K. (Heidelberg: Springer), 265–291.

[B59] Wiencke C. Clayton M. N. Gómez I. Iken K. Lüder U. H. Amsler C. D. (2007). Life strategy, ecophysiology and ecology of seaweeds in polar waters. Rev. Environ. Sci. Technol. 6, 95–126. 10.1007/s11157-006-9106-z

[B60] Wiggins A. K. Mason J. K. Thompson L. U. (2015). Growth and gene expression differ over time in alpha-linolenic acid treated breast cancer cells. Exp. Cell Res. 333, 147–154. 10.1016/j.yexcr.2015.02.020 25743093

[B61] Wulff A. Iken K. Quartino M. L. Al-Handal A. Wiencke C. Clayton M. N. (2009). Biodiversity, biogeography and zonation of marine benthic micro-and macroalgae in the Arctic and Antarctic. Bot. Mar. 52, 491–507. 10.1515/BOT.2009.072

[B62] Zanoaga O. Jurj A. Raduly L. Cojocneanu-Petric R. Fuentes-Mattei E. Wu O.. (2018). Implications of dietary ω-3 and ω-6 polyunsaturated fatty acids in breast cancer. Exp. Ther. Med. 15, 1167–1176. 10.3892/etm.2017.5515 29434704 PMC5776638

[B63] Zou Z. Bidu C. Bellenger S. Narce M. Bellenger J. (2014). n-3 polyunsaturated fatty acids and HER2-positive breast cancer: Interest of the fat-1 transgenic mouse model over conventional dietary supplementation. Biochimie 96, 22–27. 10.1016/j.biochi.2013.08.021 24012777

